# Editorial: Pain Management in Clinical and Health Psychology

**DOI:** 10.3389/fpsyg.2019.01295

**Published:** 2019-06-05

**Authors:** Gianluca Castelnuovo, Karlein M. G. Schreurs

**Affiliations:** ^1^Istituto Auxologico Italiano IRCCS, Psychology Research Laboratory, Ospedale San Giuseppe, Verbania, Italy; ^2^Department of Psychology, Catholic University of Milan, Milan, Italy; ^3^Department of Psychology, Health and Technology, Centre for eHealth and Wellbeing Research, University of Twente, Enschede, Netherlands

**Keywords:** pain management, chronic pain, clinical psychology, health psychology, psychotherapy, pain catastrophizing, placebo, nocebo

According to a recent perspective article (Castelnuovo, 2017), the exclusive medical approach in clinical field would be considered “a soul without psychology” (TIME magazine—Dec. 24, 1956). Clinical and health psychology have improved the biopsychosocial framework (Engel, 1977, 1997) ensuring a deep attention to the psychosocial issues in treating different organic and psychosomatic disorders and counterbalancing the evidence based approach with the etiquette based one in the clinician-patient relationship and communication (Kahn, 2008; Castelnuovo, 2013). Nowadays clinical and health psychology has found solutions (protocols, treatments, evidences, etc.) in any medical area: psycho-cardiology, psycho-oncology, psycho-geriatrics, psycho-pneumology, psycho-endocrinology, neuropsychology, and psychology in pain management too. “No health without mental health” (Prince et al., 2007) and “No medicine without psychology” (Castelnuovo, 2010) are two messages still effective in the daily clinical practice.

About pain management, it is important to underline that chronic pain is a relevant health problem frequently associated with psychological distress, dysfunctions in physical and social functioning, reductions in quality of life and elevated direct and indirect costs. Medical approach is useful for treating chronic pain, but effects on pain are modest (Turk et al., 2011). Psychological contributions play an important role in pain management (Castelnuovo et al.; Williams et al., 2012; Veehof et al., 2016). In fact psychological treatments are recognized as generally effective for pain (Castelnuovo et al.).

Psychological approaches in managing pain have evolved considerably and now understanding and managing the cognitions, emotions, and behaviors that accompany the situation of discomfort can actually reduce the pain intensity and the interference of pain with daily life. Psychological therapies are highly indicated both for the treatment of painful conditions and for the treatment of pain related to several neurological diseases. Similar positive results about psychotherapy efficacy were reported in specific pain disorders such as low back pain, fibromyalgia, tension-type headache and migraine, pain associated with rheumatoid arthritis, chronic abdominal pain in adolescents, chronic orofacial pain, etc. (Castelnuovo et al.).

Another important contribution of clinical health psychology in pain management is the delivery of guidelines and best practices for more integrated clinical and impactful applications. One example to replicate is the Italian Consensus Conference on Pain in Neurorehabilitation that tried to fill in the gap between theory and practice providing practical recommendations for clinicians (Castelnuovo et al.;Aloisi et al., 2016; Tamburin et al., 2016; Castelnuovo et al., 2018).

Clinical health psychology focuses also on the study of the psychological determinants in pain patients such as the role of depression, anxiety, pain-related disability, catastrophic thinking, psychological inflexibility, coping skills, beliefs, attitudes, expectations, self-efficacy, placebo, and nocebo effects, etc. Different psychological models of pain and disability (such as Fear-avoidance, Acceptance and commitment, Misdirected problem solving, Self-efficacy and Stress-diathesis models) have tried to highlight the psychological processes behind pain (McCracken and Morley, 2014).

A recent area of investigation is the study of attributions: how could comorbid symptoms worsen or improve each other? The central cognitive components of chronic pain are under investigation and could significantly influence the recovery process (Blågestad et al.).

Also measuring correctly and finely the pain phenomenon is relevant to understand the subjective experience in each patient (Boonstra et al.).

Moreover, to study the patient's life beyond pain is necessary: individuals with high perceived meaningfulness of life despite pain experienced less necessity to achieve pain control goals. Controlling pain is not necessary in order to be able to achieve non-pain goals (Crombez et al.).

Another key issue is the study of co-occurring disorders related to chronic pain, such as the sleep difficulties. ACT-based treatments for chronic pain, improving psychological flexibility, could reduce not only the level of pain, but the sleep disorders too (Daly-Eichenhardt et al.). Other ACT based protocols have been developed for pain management, for example in chronic debilitating pain for young patients (Kemani et al.) and delivering web-based psychosocial interventions (Trompetter et al.).

A typical psychological topic is the study of expectancies in pain management: optimism or pain catastrophizing can significantly shape pain experiences (Peerdeman et al.).

Further research is needed in the clinical health psychology and pain management area, also studying the role of mediators to understand the relationship between different variables, such as pain and functioning (Wicksell et al.) and the role of moderators to change in clinical psychology and psychotherapy (Holmbeck, 1997; Labus, 2007; Perz et al., 2011).

A focus on cost-analysis and cost-saving is also mandatory in the future clinical research (Giusti et al.).

## Author Contributions

GC and KS conceived of the presented idea and contributed to the final manuscript.

### Conflict of Interest Statement

The authors declare that the research was conducted in the absence of any commercial or financial relationships that could be construed as a potential conflict of interest.

## References

[B1] Aloisi A. M. Berlincioni V. Torta R. Nappi R. E. Tassorelli C. Barale F.. (2016). The role of gender, psycho-social factors and anthropological-cultural dimensions on pain in neurorehabilitation. Evidence and recommendations from the Italian Consensus Conference on Pain in Neurorehabilitation. Eur. J. Phys. Rehabil. Med. 52, 730–740. Available online at: https://moh-it.pure.elsevier.com/en/publications/the-role-of-gender-psycho-social-factors-and-anthropological-cult-3 27636563

[B2] Castelnuovo G. (2010). No medicine without psychology: the key role of psychological contribution in clinical settings. Front. Psychol. 1:4. 10.3389/fpsyg.2010.00004 21833187 PMC3153736

[B3] Castelnuovo G. (2013). 5 years after the Kahn's etiquette-based medicine: a brief checklist proposal for a functional second meeting with the patient. Front. Psychol. 4:723. 10.3389/fpsyg.2013.00723 24167493 PMC3805935

[B4] Castelnuovo G. (2017). New and old adventures of clinical health psychology in the twenty-first century: standing on the shoulders of giants. Front. Psychol. 8:1214. 10.3389/fpsyg.2017.01214 28790942 PMC5522870

[B5] Castelnuovo G. Giusti E. M. Manzoni G. M. Saviola D. Gabrielli S. Lacerenza M.. (2018). What is the role of the placebo effect for pain relief in neurorehabilitation? Clinical implications from the italian consensus conference on pain in neurorehabilitation. Front. Neurol. 9:310. 10.3389/fneur.2018.00310 29867723 PMC5968866

[B6] Engel G. L. (1977). The need for a new medical model: a challenge for biomedicine. Science 196, 129–136. 10.1126/science.847460 847460

[B7] Engel G. L. (1997). From biomedical to biopsychosocial. Being scientific in the human domain. Psychosomatics 38, 521–528. 10.1016/S0033-3182(97)71396-3 9427848

[B8] Holmbeck G. N. (1997). Toward terminological, conceptual, and statistical clarity in the study of mediators and moderators: examples from the child-clinical and pediatric psychology literatures. J. Consult. Clin. Psychol. 65, 599–610. 10.1037//0022-006X.65.4.599 9256561

[B9] Kahn M. W. (2008). Etiquette-based medicine. N. Engl. J. Med. 358, 1988–1989. 10.1056/NEJMp0801863 18463374

[B10] Labus J. S. (2007). In search of mechanisms of change in treatment outcome research: mediators and moderators of psychological and pharmacological treatments for irritable bowel syndrome. Gastroenterology 133, 702–705. 10.1053/j.gastro.2007.06.052 17681186

[B11] McCracken L. M. Morley S. (2014). The psychological flexibility model: A basis for integration and progress in psychological approaches to chronic pain management. J. Pain 15, 221–234. 10.1016/j.jpain.2013.10.014 24581630

[B12] Perz J. Ussher J. M. Butow P. Wain G. (2011). Gender differences in cancer carer psychological distress: an analysis of moderators and mediators. Eur. J. Cancer Care 20, 610–619. 10.1111/j.1365-2354.2011.01257.x 21545568

[B13] Prince M. Patel V. Saxena S. Maj M. Maselko J. Phillips M. R.. (2007). No health without mental health. Lancet 370, 859–877. 10.1016/S0140-6736(07)61238-0 17804063

[B14] Tamburin S. Lacerenza M. R. Castelnuovo G. Agostini M. Paolucci S. Bartolo M.. (2016). Pharmacological and non-pharmacological strategies in the integrated treatment of pain in neurorehabilitation. Evidence and recommendations from the Italian Consensus Conference on Pain in Neurorehabilitation. Eur. J. Phys. Rehabil. Med. 52, 741–752. 10.2147/JPR.S84646 27579583

[B15] Turk D. C. Wilson H. D. Cahana A. (2011). Treatment of chronic non-cancer pain. Lancet 377, 2226–2235. 10.1016/S0140-6736(11)60402-9 21704872

[B16] Veehof M. M. Trompetter H. R. Bohlmeijer E. T. Schreurs K. M. G. (2016). Acceptance- and mindfulness-based interventions for the treatment of chronic pain: a meta-analytic review. Cogn. Behav. Ther. 45, 5–31. 10.1080/16506073.2015.1098724 26818413

[B17] Williams A. C. Eccleston C. Morley S. (2012). Psychological therapies for the management of chronic pain (excluding headache) in adults. Cochrane Database Syst. Rev. 11:CD007407. 10.1002/14651858.CD007407 23152245 PMC6483325

